# Multimorbidity in younger deprived patients: An exploratory study of research and service implications in general practice

**DOI:** 10.1186/1471-2296-9-6

**Published:** 2008-01-28

**Authors:** Susan M Smith, Atakelet Ferede, Tom O'Dowd

**Affiliations:** 1 Department of Public Health and Primary Care, Trinity College, Dublin 2, Ireland; 2 Department of General Surgery, Waterford Regional Hospital, Ireland

## Abstract

**Background:**

Multimorbidity has been defined as the co-existence of two or more chronic conditions. It has a profound impact on both the individuals affected and on their use of healthcare services. The limited research to date has focused on its epidemiology rather than the development of interventions to improve outcomes in multimorbidity patients, particularly for patients aged less than 65 years. Potential barriers to such research relate to methods of disease recording and coding and examination of the process of care. We aimed to assess the feasibility of identifying younger individuals with multimorbidity at general practice level and to explore the effect of multimorbidity on the type and volume of health care delivered. We also describe the barriers encountered in attempting to carry out this exploratory research.

**Methods:**

Cross sectional survey of GP records in two large urban general practices in Dublin focusing on poorer individuals with at least three chronic conditions and aged between 45 and 64 years.

**Results:**

92 patients with multimorbidity were identified. The median number of conditions was 4 per patient. Individuals received a mean number of 7.5 medications and attended a mean number of GP visits of 11.3 in the 12 months preceding the survey. Barriers to research into multimorbidity at practice level were identified including difficulties relating to GP clinical software; variation in disease coding; assessment of specialist sector activity through the GP-specialist communications and assessment of the full scale of primary care activity in relation to other disciplines and other types of GP contacts such as home visits and telephone contacts.

**Conclusion:**

This study highlights the importance of multimorbidity in general practice and indicates that it is feasible to identify younger patients with multimorbidity through their GP records. This is a first step towards planning a clinical intervention to improve outcomes for such patients in primary care.

## Background

Multimorbidity has been defined as the co-existence of two or more chronic conditions in the same individual [1] and there is increasing recognition of its importance, particularly in primary care settings where many of these patients are managed [2,3]. A recent study of family practices in Canada indicated that it now represents the norm with prevalence rates of 61% in 18–44 year olds, 93% in 45–64 olds, and 98% in those over 65 years of age [4]. A sub-group of patients have higher numbers of chronic conditions or more debilitating combinations of conditions that have a high impact on their own lives and also on their utilisation of health services [5]. There has been limited research into multimorbidity and most has focused on the exploration of large patient databases in an attempt to understand its full impact [6]. Experimental research on interventions to improve the management of patients with multimorbidity has been lacking, though a Cochrane Review of such interventions is ongoing [7]. Several barriers to the introduction of an intervention for multimorbidity exist in most healthcare systems. These include identifying patients using existing clinical software, which uses single disease coding systems, the estimation of the process of care, and polypharmacy issues, all of which make management of these patients more complex for primary care practitioners.

While primary care has expressed most interest in multimorbidity, there has been limited assessment of the workload of managing these patients in their communities. This is particularly important for younger patients with multimorbidity who may attend multiple specialists and need coordination of their care by their general practitioner. In Ireland and many other countries, patients aged 65 and over can be referred to geriatricians who have maintained a generalist physician approach and work with multidisciplinary teams. The increasing sub-specialisation of physicians has led to a situation where there are no equivalent services for those aged less than 65.

This paper presents results from an exploratory study of multimorbidity in two general practices in deprived settings in Dublin designed to inform a larger study of an intervention to improve outcomes in patients with multimorbidity. For this exploratory study we focused on younger patients with multimorbidity, specifically those with more complex health needs having three or more chronic conditions. We aimed to document the health services provided to these patients compared to individuals with a single chronic conditon. We also aimed to identify and describe the barriers encountered in conducting research into multimorbidity in primary care settings.

## Methods

### Setting

The survey was conducted in two general practices in Dublin: Inchicore Medical Centre (IMC) and Mary Mercer Health Centre (MMHC), where two of the authors practice as GPs. IMC has three full-time equivalent GPs and one practice nurse. MMHC has two full-time equivalent GPs and one practice nurse. Both practices are based in deprived urban areas, are fully computerized and use the International Classification for Primary Care (ICPC-2) coding of diseases. Primary care in Ireland is funded through a mixed set of payments with the poorest 28% of the population being eligible for free primary care based on a capitation payment system (the General Medical Service (GMS) Scheme). Specialist outpatient care is available to all without payment but there are long waiting times for routine appointments. Those not eligible for free GP care pay for both visits and medications with each visit costing on average €50.

### Patients

In an effort to identify patients with higher levels of multimorbidity, who present particular management problems for primary care, we focused on younger, more socio-economically deprived patients with three or more chronic conditions.

### Inclusion and exclusion criteria

Patients with three or more chronic conditions, aged between 45–64 years and with eligibility for the GMS scheme were included. Chronic medical conditions were counted and compiled based on World Health Organization's (WHO) definition of chronic conditions [8] and O'Halloran et al's definition of chronic conditions for primary care [9]. We excluded the following groups of patients, those aged under 45 and over 65 year olds, those not eligible for the GMS scheme and those with two or less chronic conditions. Records of patients with three or more chronic conditions were further checked by a clinician researcher (AF) and excluded from the study if they did not fulfill the inclusion criteria.

We also identified a small comparator group of individuals within the same age and socio-economic grouping who had only one chronic condition to provide a comparator group. This was only done in the IMC practice, as it was not possible in the MMHC practice to use the practice clinical software system to identify individuals age 45–64 with one chronic condition and would have involved extensive individual patient record searching which was not possible due to time and funding constraints.

### Data collection

Data collection was carried out during May 28–June 22, 2007. One clinical researcher (AF) reviewed the electronic medical records and completed the data extraction questionnaire. The selection process of patients is illustrated in Tables 1 &2. The list of variables included age, sex, marital status, employment, list of chronic conditions and list of current medications. Data on GP visits, practice nurse visits, telephone consultations, house calls, hospital admissions, in-patient days and hospital visits over the last twelve months preceding the survey were also retrieved. The hospital visits included all visits other than admissions, i.e., OPD, A&E, dietician, physiotherapist, speech and language visits, endoscopies, colonoscopies, angiography, etc. The approach for data extraction in the two practices varied as they had different computer databases. In IMC we also collected equivalent data on a comparator group of 42 patients, also aged 45–64 with a single chronic condition.

**Table 1:** Characteristics of study population with multimorbidity

	N = 92
Sex	
Male	43 (47%)
Female	39 (53%)

Number of Chronic Condition (Mean ± SD)	4 ± 1.1
Number of Current Medications (Mean ± SD)	7.5 ± 3.3
GP Visit/12 months (Mean ± SD)	11.7 ± 7.1
Practice Nurse Visit/12 months (Median ± IQR)	1 (0–4)
Hospital Visit/12 months (Mean ± SD)	3.3 ± 3

**Table 2:** Prevalence of common chronic conditions among study population

DIAGNOSIS (ICP-2 Code)	INCHICORE N (%)	MARY MERCER N (%)
Lipid Disorder (T93)	29 (11.4)	22 (18.5)
Hypertension (K86)	25 (9.8)	18 (15.1)
Depressive Disorder (P76)	18 (7.1)	5 (4.2)
NIDDM ^¶ ^(T90)	13 (5.1)	12 (10.1)
COPD ^Π ^(R95)	11 (4.3)	9 (7.6)
Asthma (R96)	10 (3.9)	8 (6.7)
Acute Myocardial Infarction (K75)	9 (3.5)	0 (0)
IHD ^¥ ^without Angina (K76)	6 (2.4)	1 (0.8)
IHD ^¥ ^with Angina ((K75)	9 (3.5)	4 (3.4)
Cardiovascular Disease Other (K99)	3 (1.1)	5 (4.2)
Chronic Alcohol Abuse (P15)	6 (2.4)	5 (4.2)
Hiatus Hernia (D90)	6 (2.4)	0 (0)

^¶ ^NIDDM, Type 2 Diabetes (Non Insulin Dependent Diabetes)

^Π ^COPD, Chronic Obstructive Pulmonary Disease

^¥ ^IHD, Ischemic Heart Disease

### Statistical Analyses

Data were entered onto Excel and then transferred onto the statistical software JMP In version 4 for analysis. Univariate analysis included numbers and frequencies. Bivariate analysis included Fischer's and student's t-Test for comparison of proportions and means respectively. The analysis focused on a comparison of multimorbidity patients in both practices and a comparison between multimorbidity patients and comparator patients with a single chronic condition in the IMC practice only.

### Process evaluation

The researcher kept a diary of all problems encountered during the data collection phase with a view to identifying and describing barriers encountered.

## Results

We identified 62 eligible patients in IMC from a possible 267 GMS eligible individuals aged 45–64 (23%). In the MMHC the GP software could not specifically identify GMS patients aged 45–64 and we identified 30 eligible patients from a total GMS list of 685 (all ages). There were no significant differences between the eligible patients from both practices other than gender balance (IMC 52% male and MMHC 34% male). The 92 patients identified in both practices (Table 1) had a median (IQR) age of 58 (52–60) years. Data on marital status was only recorded for 22 patients (24%) and equal numbers of those were either married or single. The mean number of chronic conditions per patient was 4. Patients with multimorbidity received an average of 7.5 medications. The mean number of GP visits over the last 12 months preceding the survey was 11.7. Hospital visits in the previous 12 months ranged from 0–12 for IMC and 0–4 for MMHC. Data on hospital activity relating to both diagnoses, medications and hospital admissions was incomplete in both practices.

A total of 85 different diagnoses were identified. The most common chronic conditions were lipid disorder, hypertension, depressive disorder, NIDDM, COPD, asthma and acute myocardial infarction (Table 2).

Patients with multimorbidity at the IMC practice (n = 62) were significantly more likely to attend the GP than the comparator group of patients with a single chronic condition (n = 42) in the previous 12 months (Table 3).

**Table 3:** Comparison between single morbidity and multimorbidity, Inchicore Medical Centre only

Characteristics	Single Morbidity N = 42	Multimorbidity N = 62	P value
Sex			
Female, N%	20 (47.6%)	30 (48.4%)	0.939
Male, %	22 (52.4%)	32 (61.6%)	
Age, Mean ± SD	53.7 ± 5.73	56.0 ± 5.3	0.040
GP Visit/12 months, Mean ± SD	7.2 ± 6.5	12.6 ± 7.5	0.0003
Number of current medication, Mean ± SD	2.3 ± 2.3	7.3 ± 3.4	<0.0001

### Barriers to research into multimorbidity at practice level

#### 1. Issues relating to record keeping and disease coding

Both practices used electronic records only and there was variation in the quality and type of data recorded. A direct search for patients with multiple chronic conditions was not possible in either system and we had to cross reference lists generated for single common chronic conditions. The software support services for the practices plan to develop a system to facilitate searching for patients with multimorbidity.

The recording of demographic data, particularly marital status, employment, nationality and ethnicity was not done consistently in the practices.

Both current and past medical conditions were sometimes coded together on the database, e.g. NIDDM and Cholelithiasis (for which the patient was operated years ago). Consequently we excluded 28 patients from the original 90 identified in IMC leaving 62 individuals with multimorbidity. Not having a list of active diseases and regular data update leads to an overestimation bias unless records are carefully screened by a researcher with clinical knowledge.

Another example of variation between the two practices related to coding for lipid disorders with some patients on lipid lowering medication not being coded as having a lipid disorder even though it is classified as a chronic disease. The practitioners may regard many individuals having treatment with lipid lowering medication as having risk factor management rather than chronic disease management. The ICPC coding of hypertension was also unclear. Although the ICPC classifies hypertension as 'uncomplicated' or 'complicated', both practices coded all hypertension as uncomplicated. Comprehensive chart searching also indicated inconsistencies in disease coding with some individuals being prescribed repeat inhalers though not being coded as having asthma or COPD. Additional diagnoses were also retrieved from other sources such as hospital letters. Therefore it is possible we might have missed patients with multiple chronic conditions and underestimated the extent of multimorbidity.

#### 2. Examining the process of primary care delivery

Assessment of primary care activity requires a consideration of the different consultation types. Although telephone consultations and house calls were recorded it was difficult to differentiate the two from face to face GP consultations, with a quick review of the computer record. Thus all the notes were reviewed and screened for content, which was time consuming and increased the administrative burden. It was also unclear whether the telephone consultation involved a GP or practice nurse. In one of the practices, the staff indicated that consultations involving house calls or telephone consultations were rarely recorded. This underestimates the extent of consultation activity and its workload implications.

#### 3. Multiple sources of information

There was variation in recording of information from the specialist sector with one practice routinely scanning all letters while the other kept paper copies and wrote summaries into patient records. Thus it was not possible to record the number of hospital admissions, in-patient days and general hospital visits for 22 of 30 patients in MMHC. There is no single patient identifier in the Irish healthcare system and this work highlights the potential benefit of such an identifier in relation to comprehensive assessment of care delivery in all sectors.

## Discussion

### Study findings: prevalence and workload implications

This exploratory survey has highlighted how common multimorbidity is, even in a younger patient group and indicates the degree of polypharmacy and the increased workload involved in managing these patients in primary care. While it has confirmed the feasibility of identifying individual multimorbidity patients through their GP many limitations are evident from the data which is collected for service clinical care and not research purposes. Our results are similar to other larger international studies, which have also highlighted the burden of multimorbidity [1,4,10]. These studies have found similar types of chronic conditions though our sample has fewer cases with rheumatological conditions, which may relate to disease coding within the practices. They have also highlighted the impact of multimorbidity on the process and complexity of care. This will vary between healthcare systems depending on the balance between primary and specialty care provision within the system.

### Barriers identified

Several barriers to conducting multimorbidity research and interventions in primary care settings were identified. These included issues in relation to record keeping and disease coding; estimating primary care workload and the presence of multiple sources of information from within and beyond primary care records.

Variations in the prevalence of chronic conditions between the two practices may relate to variations in disease prevalence in the catchment areas or to clinical variations in diagnosis between practitioners in the two centers. Both practices used the international ICPC-2 classification of diseases, which is useful for consistency and comparison with similar studies though limitations with the coding system were also identified. Data quality could be improved further by recording all demographic factors, updating records regularly, making patient notes more detailed, scanning all letters from hospitals and recording all patient consultations separately and consistently including GP visits, practice nurse visits, telephone consultations and house calls.

### Study limitations

A limitation of this study in relation to exploring the full impact of multimorbidity was the fact that it involved an analysis of patient records only. Ideally the impact of multimorbidity needs to be measured using a multimorbidity index [11] such as the Cumulative Illness Rating Scale (CIRS)[12]. This takes into account the number of medical problems and weights them according to their severity for an accurate assessment of the impact on the patient's health. This would require direct patient review including a detailed medical history for each condition and the use of a validated scoring manual [12]. Direct patient contact would also allow an understanding of the needs and preferences of patients with multimorbidity and of its impact on their quality of life and the impact of multimorbidity on family and carers [13,14].

## Conclusion

This exploratory study has described the burden of multimorbidity among younger deprived patients in general practice. Clinical complexity and polypharmacy are major drivers of GP workload which have to be taken into account in delivering a clinical intervention to improve outcomes for younger patients with multimorbidity in primary care.

## Competing interests

The author(s) declare that they have no competing interests.

## Authors' contributions

SMS and TOD conceived of the study. AF collected and analysed the data. SMS, AF and TOF drafted the manuscript. All authors read and approved the final manuscript.

## Pre-publication history

The pre-publication history for this paper can be accessed here:


